# Association of *FLG* mutation with tumor mutation load and clinical outcomes in patients with gastric cancer

**DOI:** 10.3389/fgene.2022.808542

**Published:** 2022-08-15

**Authors:** Fu Yicheng, Liu Xin, Yu Tian, Liu Huilin

**Affiliations:** ^1^ Department of Geriatrics, Peking University Third Hospital, Beijing, China; ^2^ Department of Gastroenterology, Peking University Third Hospital, Beijing, China; ^3^ Graduate School, Chinese Academy of Medical Sciences and Peking Union Medical College, Beijing, China

**Keywords:** filaggrin, stomach adenocarcinoma, TCGA, prognostic model, tumor microenvironment FLG mutation in gastric cancer

## Abstract

**Background:** Stomach adenocarcinoma (STAD) is one of the most frequently diagnosed cancers in the world with a poor prognosis due to genetic heterogeneity. The present study aimed to explore potential prognostic predictors and therapeutic targets that can be used for STAD treatment.

**Methods:** We collected relevant data of STAD patients from the Cancer Genome Atlas (TCGA), including somatic mutation, transcriptome, and survival data. We performed a series of analyses such as tumor mutational burden (TMB), immune infiltration, and copy number variation (CNV) analysis to evaluate the potential mechanism of filaggrin (*FLG*) mutation in gastric cancer. Gene ontology (GO), Kyoto Encyclopedia of Genes and Genomes (KEGG) analysis, and gene set enrichment analysis (GSEA) were performed for annotation of differentially expressed genes (DEGs). The STRING online database was used to construct the protein–protein interaction (PPI) and ceRNA network and hub genes were identified. Univariate and multivariate Cox regression analyses were used to determine the effect of selected DEGs on tumor prognosis.

**Results:** The *FLG*-mutant group (*FLG*-MT) showed a higher mutation load and immunogenicity in gastric cancer. GO and KEGG analyses identified and ranked unique biologic processes and immune-related pathway maps that correlated with the *FLG*-mutant target. GSEA analysis showed that several tumorigenesis and metastasis-related pathways were indeed enriched in *FLG*-mutant tumor tissue. Both cell cycle–related pathways and the DNA damage and repair associated pathways were also enriched in the *FLG*-MT group. The *FLG* mutations resulted in increased gastric cancer sensitivity to 24 chemotherapeutic drugs. The ceRNA network was established using Cytoscape and the PPI network was established in the STRING database. The results of the prognostic information further demonstrated that the OS and DFS were significantly higher in *FLG* mutation carriers, and the *FLG* gene mutation might be a protective factor.

**Conclusion:** The multiple molecular mechanisms of the *FLG* gene in STAD are worthy of further investigation and may reveal novel therapeutic targets and biomarkers for STAD treatment.

## Introduction

Gastric cancer is one of the most common gastrointestinal malignancies and still constitutes a health burden worldwide which was responsible for over one million new cases of mortality globally (Sung et al., 2021). Stomach adenocarcinoma (STAD) constitutes approximately 95% of GC cases, as a well-recognized heterogeneous type, its rate of 5-year survival is less than 30% for the advanced stage due to lack of effective therapeutic modalities (Ajani et al., 2017). Many DNA alterations have been detected in STAD, and accumulating evidence has demonstrated a crucial role in genetic correlation research (Ramezankhani et al., 2021). Recently, the genomic mechanisms of STAD have been widely studied, and many advanced STAD patients achieved better life expectations through the progression of targeted therapies and the chemotherapeutic drug optimization. The therapeutic backbone of metastatic STAD remains molecularly targeted therapies, which include *HER2*-targeting agents, anti-angiogenic agents, and epidermal growth factor receptor (*EGFR*) inhibitors (Alsina et al., 2019). However, several studies have failed to find the survival benefit of specific new innovative agents due to the marked multidrug-resistant phenotype of STAD, only three targeted therapeutics showed modest OS benefit in phase III trials: trastuzumab, ramucirumab, and lapatinib (Mizrak et al., 2017). However, negative trials with targeted agents have significantly outnumbered the positive trials in STAD in the past few years, and the cause of the genomic alterations in tumor growth and drug resistance are not fully known. For example, in the GATSBY trial, compared with paclitaxel or docetaxel as second-line treatment, trastuzumab showed no OS benefit (*p* = 0.86) and was not recommended as routine second-line treatment for *HER2*-positive advanced GAC (Liguigli et al., 2014). The two-phase III first-line and second-line trials of lapatinib both observed the absence of the primary end-point of OS advantage (Satoh et al., 2014; Hecht et al., 2016). The inherent genomic instability may give rise to a weak and inefficient response to cancer drug therapies, eventually leading to tumor progression and treatment failure (Russi et al., 2019). Thus, developing more specific and reliable biomarkers for clinical trials is essential for understanding the mechanisms of drug action in STAD therapy.

Recently, bioinformatics has become an effective tool for screening significant genetic variations that occur in carcinogenesis and offers a great promise for improved diagnosis, prognosis, treatment selection, and surveillance for cancer patients. A previous study used bioinformatics to predict the DEGs of STAD and its enriched pathways and screened and evaluated some hub genes to provide some ideas and references for the early diagnosis and treatment of STAD at the molecular level. For example, upregulation of *COL3A1*, *COL1A2*, *BGN*, and *THBS2* were found to significantly reduce the survival time of STAD patients (Qiu et al., 2020). *FN1*, *SPARC*, and *SERPINE1* were highly expressed and significantly related to a poor prognosis of STAD (Li et al., 2019). The high expressions of *PER1* and *NR1D1* were not only associated with poor OS, progression-free survival, and disease-free survival, but also associated with immune infiltration in STAD patients (Huang et al., 2021). RNA binding protein genes such as *PTBP1*, *PPIH*, *SMAD5*, *MSI2*, *RBM15*, *MRPS17*, and *ADAT3* were identified to be prognosis-related in STAD patients, the regulatory network and functional study showed *MRPS17* and *PTBP1* could reduce the number of infiltrated immune cells. The complement component 3a receptor 1 (*C3AR1*) was proven to promote the polarization of M2 macrophages and T-cell exhaustion, leading to the immune escape of STAD and high expression of the *C3AR1* gene is correlated with a poor prognosis (Li et al., 2021). In addition, in combination with bioinformatics, a prognostic model has been developed and proven capable of predicting prognosis of STAD patients (Ye et al., 2020). Nevertheless, the consequences of genomic alterations on tumor growth and drug resistance remain largely unexplored. The potential reason is most likely a combination of complex modes of inheritance span of different tumor stages and lack of specific target biomarkers. So far, no biomarker has been shown to be accurate enough to diagnose or predict the prognosis of STAD. Accordingly, as one of the emerging frontiers of STAD carcinogenesis and therapeutic target exploration, in-depth informatics investigation with larger sample sizes and fine-grained understanding of new genetic loci are helpful to identify more robust and reliable genetic biomarkers.

In this study, we systematically analyzed the somatically mutated genes of STAD based on TCGA database and screened out the filaggrin (*FLG*) gene for further investigation. The *FLG* gene could be an important candidate for STAD, which coincides with findings in another study (Wang et al., 2020). Filaggrin (*FLG*) protein, which is known as a filament-aggregating protein, is important for the formation of the stratum corneum and was proven to play a key role in the maintenance of an optimal skin, oral, and cervical mucosa barrier. Its monomer can combine with the keratin filaments as a matrix protein and results in aggregation of the keratinocytes. These keratinocytes act as a matrix in the stratum corneum. A previous study has shown that *FLG* gene mutations possibly bring about a greater susceptibility to Epstein–Barr virus (EBV)–associated gastric carcinoma (Kuang et al., 2016). This may be related to the fact that about 9% of gastric cancers harbor EBV infection. The deletion mutations of the *FLG* gene are also prevalent in Asian populations, so making related genetic research valuable (Salama et al., 2021). However, the precise mechanisms underlying its development are still unclear as little is known about the role of the *FLG* gene. Thus in this study, we performed an in-depth investigation into the functional roles of the *FLG* gene in STAD. According to the mutation status of the *FLG* gene, patients were divided into *FLG*-mutant (MT) and *FLG*-wild-type (WT) groups. We found that the *FLG*-MT gastric cancer patients had showed a higher mutation load and immunogenicity. In addition, we explored the differences in pathway activation between the *FLG*-MT gastric cancer patients and *FLG*-WT patients through functional enrichment analysis to explain the effect of *FLG* on the tumor microenvironment (TME). Next, we obtained insights into prognosis evaluation, protein and ceRNA interaction networks, immune infiltration, and anticancer drug sensitivities, which may provide valuable references for diagnosis, targeted drug research, and prognosis evaluation of STAD.

## Materials and methods

### Data downloads

The masked somatic mutation data of STAD patients used in this study were retrieved from the TCGA GDC database (http://portal.gdc.cancer.gov/). Patients with a pathologic diagnosis of stomach adenocarcinoma were included. The data were preprocessed by VARSCAN software, and visualizations of somatic mutations were carried out in R (Foundation for Statistical Computing, Vienna, Austria) using the package Maftools (Mayakonda et al., 2018). The gene expression data (FPKM value) of the patients’ RNA sequencing were downloaded and converted to the TPM value, and the lncRNA and mRNA associations were then established. In addition, the clinicopathological features and prognosis of the STAD patients, such as gender, age, malignant stage, TNM stage, and MSI value, all these data were obtained from the UCSC Xena (http://xena.ucsc.edu/). We used the Tumor Immune Dysfunction and Exclusion (TIDE) algorithm (http://tide.dfci.harvard.edu) to predict the response to immunotherapy of each sample (Jiang et al., 2018).

### Copy number variation analysis

To analyze the copy number variations (CNVs) of *FLG* genes in TCGA-STAD patients, the masked copy number segment data were downloaded using the TCGAbiolinks R package (version 2.6.12). GISTIC 2.0 was used to conduct GenePattern5 analysis of downloaded CNV fragments (Reich et al., 2006). We use default settings in the GISTIC 2.0 analysis with the exception of several parameters (e.g., the confidence coefficient was 0.99; X chromosomes were not excluded prior to analysis). Finally, the results of GISTIC 2.0 analysis were visualized using the Maftools package of R software (Mayakonda et al., 2018).

### Calculation and correlation analysis of somatic mutation and tumor mutation load fraction

Tumor mutational burden (TMB) in this study was defined as the number of somatic synonymous mutations per megabase in each tumor sample, with silent mutations excluded. The Wilcoxon matched-pairs signed-rank test was used to compare TMB values between *FLG* mutation and non-mutation groups.

### Identification of differentially expressed genes (DEGs) and clinical correlation analysis

To analyze the effect of *FLG* mutation on tumorigenesis in STAD patients, the samples in the TCGA database were divided into a mutation group and a non-mutation group according to the *FLG* mutation situation. The DEGs between the two groups were determined using the DESeq2 package in R (Love et al., 2014). The cut-off criteria for statistical significance were a log-fold change (FC) of greater than 1 and a *p*-value of less than 0.05. Visualizations of differentially expressed genes such as volcano plots and heatmaps were generated using standard R packages.

### Functional enrichment analysis

The GO analysis serves as a bioinformatics tool that provides structured annotations, including biological processes (BPs), molecular functions (MFs), and cellular components (CCs), for genes and gene products. KEGG (http://www.genome.jp/) is a widely used database storing information about genomes, biological pathways, diseases, and drugs. Enrichment plots of gene signatures were generated using the R package clusterProfiler (Yu et al., 2012), and FDR critical value of less than 0.05 was considered to indicate a statistically significant difference. To investigate the differences in biological processes between different groups, the enrichment analysis was performed using GESA (Hanzelmann et al., 2013). GSEA is a statistical method to assess whether a priori defined set of genes shows statistically significant concordant differences between two different biological statuses (Subramanian et al., 2005). GSEA analysis of the gene expression profiling dataset of TCGA-STAD patients was implemented using the clusterProfiler package. C2. all.v6.2. symbols.gmt was selected as the reference gene set. False discovery rate < 0.1, and *p*-value < 0.05 were set as the cut-off criteria.

### Comparison of biological functions and immune estimation scores

We further analyzed the correlation between different subgroups and some biological related processes. The immune and stromal scores were evaluated by applying the ESTIMATE algorithm using the estimate R package (R version 3.5.3) (Yoshihara et al., 2013). The scores were used to reflect the level of immune cell and stromal cell infiltration of tumor tissue. The Mann–Whitney U-test was used to compare the infiltration levels of immune cells between two groups.

### Protein–protein interaction network construction

The PPI information available in the STRING network in the STRING database (http://string-db.org, version 10) is useful for predicting physical and functional interactions (Szklarczyk et al., 2019). All DEGs were mapped to the STRING database, and the interactions with reliability scores of more than 0.4 were selected to analyze the relationship of the DEGs. Cytoscape v3.7.2 was used to select the key nodes with the strongest connectivity for visualizing molecular interaction networks (Shannon et al., 2003). The MCODE plugin in Cytoscape 3.7.2 was used to identify the most densely connected region in the PPI based on vertex weights, which could identify hub genes in the PPI network.

### Construction of ceRNA networks

We further retrieved experimentally validated miRNA–mRNA interactions from the miRTarBase. Based on core mRNAs obtained from PPI interaction analysis, miRTarBase was utilized to predict possible regulatory miRNA, and further predict related lncRNA. The alluvial diagrams of the co-expression network with the overlapped lncRNA–miRNA–mRNA relationships were generated using the R package ggalluvial.

### Sensitivity analysis of anticancer drugs

Genomics of Drug Sensitivity in Cancer (GDSC; https://www.cancerrxgene.org/) is a public database for tumor molecular therapy and mutation exploration (Vanden et al., 2018). The R package pRRophetic was used for downloading cell line gene mutation data and IC50 values of different anticancer drugs and analyzing the correlation between *FLG* gene mutation patients and the sensitivity of different anticancer drugs.

### Validation of clinical prediction models

The relationship between clinicopathological and prognostic features (overall survival OS) and *FLG* gene mutations of STAD patients in TCGA was analyzed with the logistic regression and receiver operating characteristic (ROC) methods. The Harrell consistency index (C-index) of *FLG* expression was based on the best separation. The diagnostic ROC curve was used to explore the prognostic or predictive accuracy of each characteristic underlying the area under the curve (AUC). The Kaplan–Meier curve was used to estimate the effects of *FLG* on the overall survival of STAD patients.

### Statistical analysis

All statistical analyses were performed as the means ± standard deviation. The R software (version 4.0.2) was utilized to measure the data. Quantitative data that were not normally distributed were evaluated using the Mann–Whitney U-test, and Student’s t-test was used for normally distributed data. For analysis of relations between categorical variables, we used the chi-squared test or Fisher’s exact test when appropriate. The correlation coefficients among different genes were calculated by Pearson correlation analysis. Survival analysis was estimated using R package survival and survival differences were determined by Kaplan–Meier analysis, the log-rank test was used to compare OS among groups. R software with the package pROC was used to produce the ROC curve, the area under the curve (AUC) as a measure of accuracy. All tests were two-sided with a significance level of *p* < 0.05.

## Results

### Association between the *FLG* status and clinical characteristics

**FIGURE 1:**
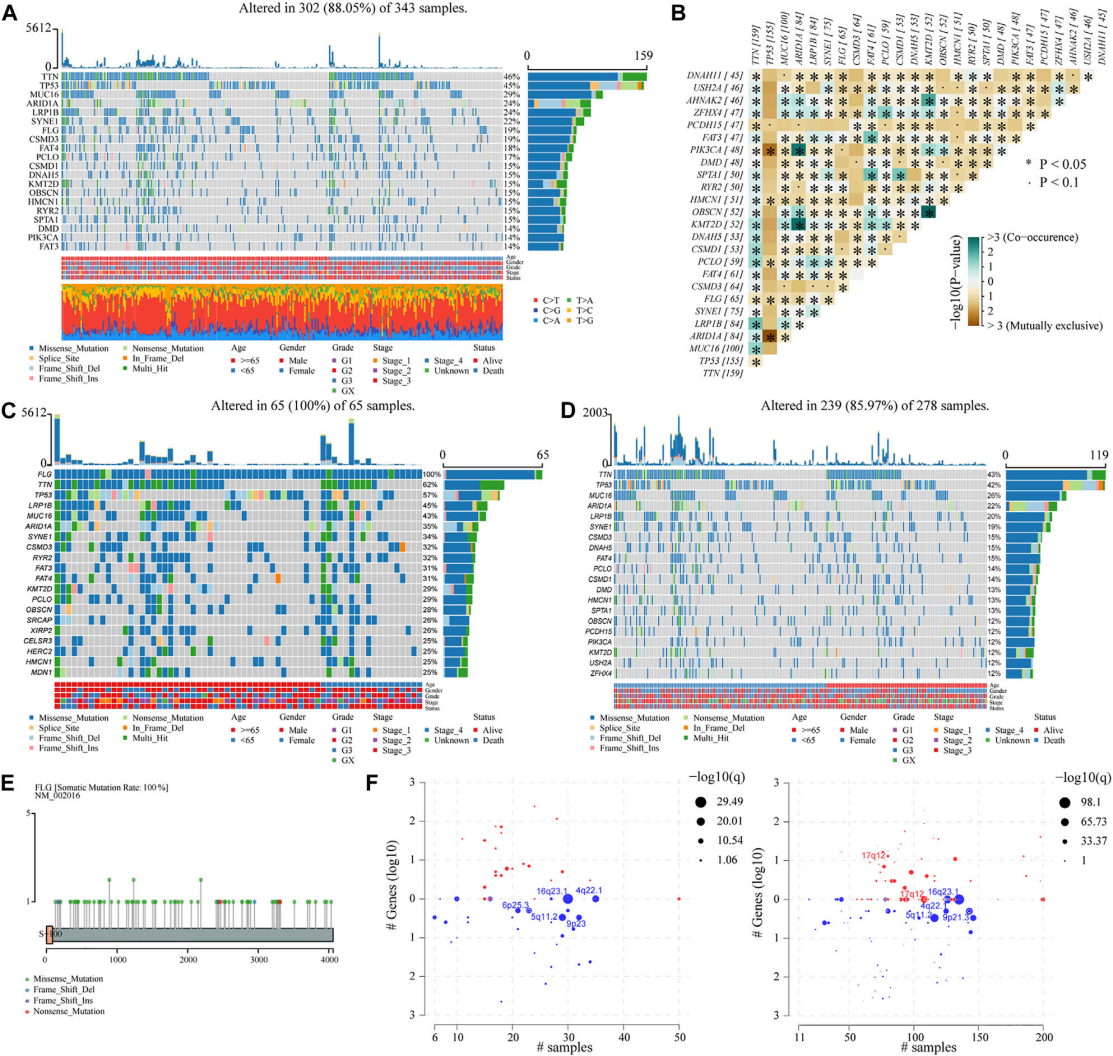
Correlation of clinical characteristics of STAD patients with *FLG* gene mutation. **(A)** Waterfall plot for all STAD patients in the study. Types of mutations were classified according to different categories, the majority of which were missense mutations. The C> T mutation is the most common SNV. The corresponding TMB values of specific tumor samples and top 30 ranked mutant genes are also shown. **(B)** Correlation analysis of different mutant genes. **(C,D)** Top 30 most frequently mutated genes in *FLG*-mutant and non-mutant groups. The left side is the *FLG*-mutant group, the right side is the non-mutant group. Genes are sorted by mutational frequency, and samples are sorted and ordered according to non-synonymous mutational load. The legend earlier shows the mutation load. Age, gender, grade, stage, and OS status are noted in order. **(E)** Distribution diagram of amino acid variation of FLG proteins in the TCGA-STAD dataset, missense mutation is the main form. **(F)** TCGA-STAD samples with available CNV data were analyzed using GISTIC 2.0 software and visualized using Maftools package.

Somatic genetic alterations data on STAD patients were downloaded from the TCGA database and analyzed as previously described (see Table 1). The waterfall plot for all STAD patients in the study showed that the top ten most mutated genes are *TTN*, *TP53*, *MUC16*, *ARIDIA*, *LRPIB*, *SYNE1*, *FLG*, *CSMD3*, *FAT4*, and *PCLO*, and 19% of the patients carried *FLG* mutation. Missense mutations accounted for the majority of mutation types of STAD patients, with C>T being the most common single-nucleotide variant (SNV) (Figure 1A). Meanwhile, correlation analysis showed that *FLG* had co-occurrence with *LRP1B* and *RYR2* (*p* < 0.05), and tended to be mutually exclusive with *TTN*, *TP53*, and *MUC16* (*p* < 0.05). (Figure 1B). TCGA-STAD patients were allocated into two groups based on the *FLG* mutation status, the *FLG*-mutant group (*FLG*-MT) (Figure 1C) and *FLG*-wild-type group (*FLG*-WT) (Figure 1D). The left side is the *FLG*-MT group, and the right side is the *FLG*-WT group. Genes are sorted by the mutational frequency, and samples are sorted and ordered according to the non-synonymous mutational load. The top 30 most frequently mutated genes of *FLG*-MT and *FLG*-WT groups are shown in the waterfall plot. The amino acid changes of the *FLG* gene are highlighted in Figure 1E, the missense mutation appeared to be the major form of mutations in all STAD patients. Meanwhile, based on *FLG* mutation levels, the CNV data of TCGA-STAD patients were divided into the mutant group and the non-mutant group. As shown in Figure 1F, the CNV levels of multiple genes have changed significantly.

**TABLE 1:** The baseline patient data of STAD in TCGA database.

Variables	All patients	*FLG*-WT	*FLG*-MT	*p* Value
	(*n* = 343)	(*n* = 278)	(*n* = 65)	
Age				0.014*
<65	144 (42.0%)	126 (45.3%)	18 (27.7%)	
≥65	199 (58.0%)	152 (54.7%)	47 (72.3%)	
Gender				0.761
Female	119 (34.7%)	98 (35.3%)	21 (32.3%)	
Male	224 (65.3%)	180 (64.7%)	44 (67.7%)	
Grade				0.01*
G1 & G2	134 (39.1%)	99 (35.6%)	35 (53.8%)	
G3 & GX	209 (60.9%)	179 (64.4%)	30 (46.2%)	
T				1
T1 & T2	88 (25.7%)	71 (25.5%)	17 (26.2%)	
T3 & T4 & TX	255 (74.3%)	207 (74.5%)	48 (73.8%)	
M				0.079
M0	306 (89.2%)	244 (87.8%)	62 (95.4%)	
M1 & MX	37 (10.8%)	34 (12.2%)	3 (4.6%)	
N				0.09
N0 & N1	192 (56.0%)	149 (53.6%)	43 (66.2%)	
N2 & N3 & NX	151 (44.0%)	129 (46.4%)	22 (33.8%)	

### Relationships between the *FLG* mutation status and biological characteristics and mutation load in STAD

**FIGURE 2:**
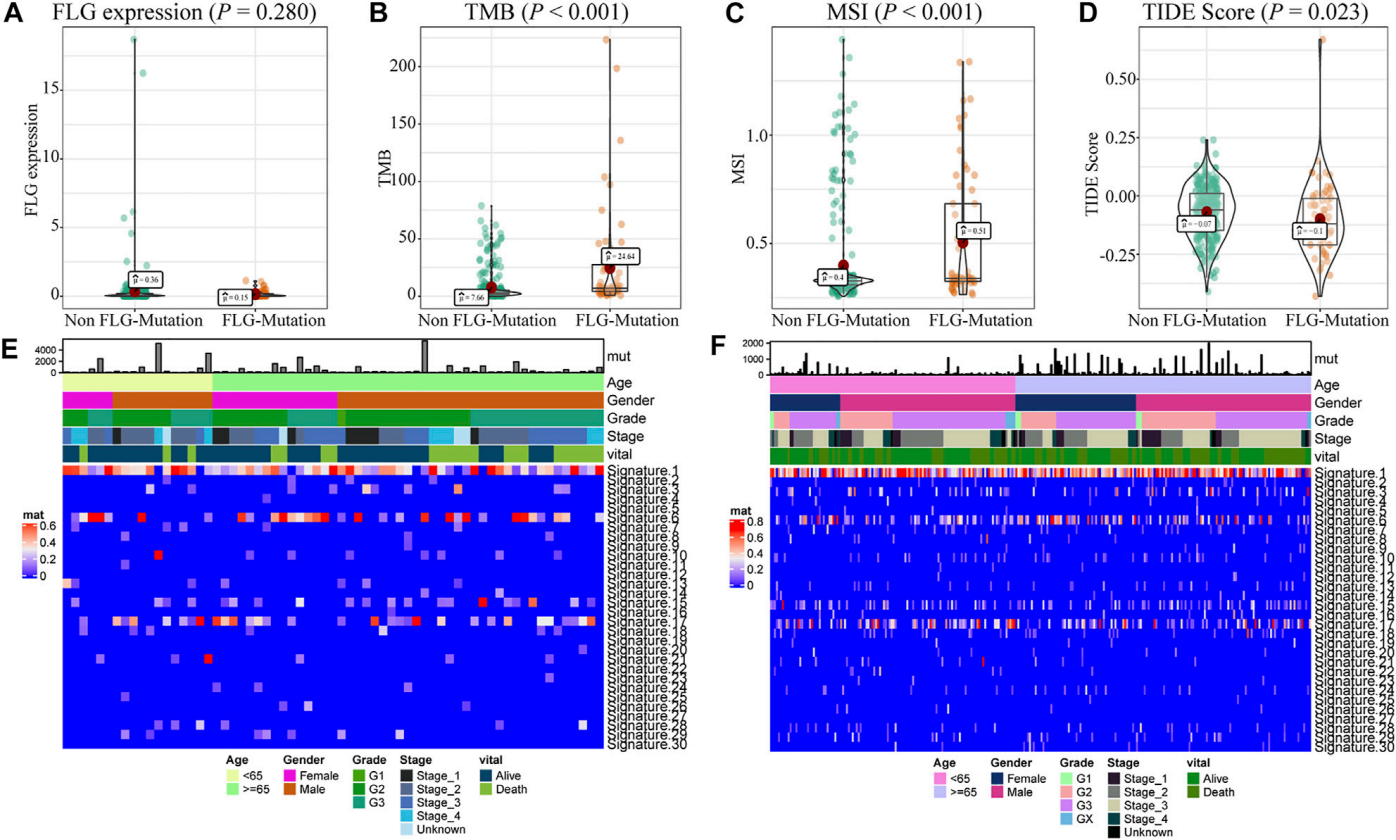
Biological characteristics of *FLG* gene mutations in STAD patients. **(A)** There were no significant differences in gene expression levels between patients with and without *FLG* mutations. (*p* = 0.280). **(B)** TMB was significantly higher in patients with *FLG* mutation (*p* < 0.001). **(C)** MSI was significantly higher in patients with *FLG* mutation (*p* < 0.001). **(D)** Patients with *FLG* mutation had a significantly lower TIDE score than those in the non-mutant group (*p* = 0.023). The lower the TIDE score, the better the effect of immunotherapy. **(E)** Cosmic signature thermogram analysis of *FLG*-mutant patients in the TCGA dataset, the corresponding clinical features of the patients are shown earlier. **(F)** Cosmic signature thermogram analysis of non-mutant patients in the TCGA dataset.

**FIGURE 3:**
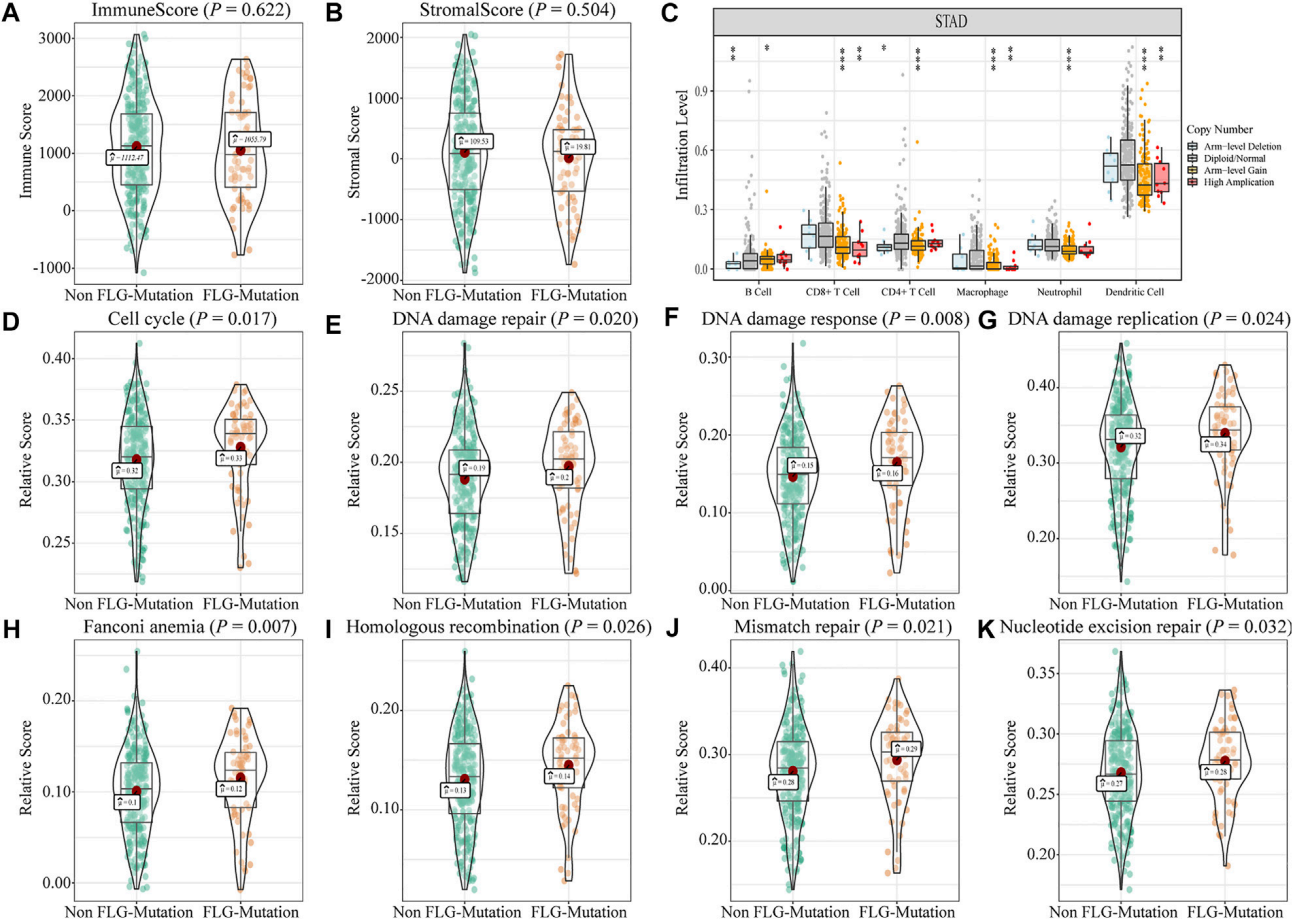
Effects of *FLG* gene mutations on immunological characteristics in TCGA-STAD datasets. **(A)** In TCGA-STAD datasets, the immune scores in the *FLG*-mutant group were unremarkable compared to those in the non-mutant group. (*p* = 0.622). **(B)** Matrix score in the *FLG*-mutant group was unremarkable compared to that in the non-mutant group. (*p* = 0.504). **(C)** Immune cell infiltration was analyzed using the TIMER website. The significant differences were observed in the immune cell infiltration between *FLG*-mutant and non-mutant groups. **(D–K)** Biological functions were significantly different between mutant and non-mutant groups (*p* < 0.05), including cell cycle–related pathways, DNA injury repairment-related pathways.

We analyzed the effects of *FLG* gene mutations on different biological characteristics. The *FLG* gene expression levels did not differ significantly between *FLG*-mutant and non-mutant groups (*p* = 0.280), as shown in Figure 2A. The TMB value (Figure 2B, *p* < 0.001) and MSI value (Figure 2C, *p* < 0.001) were elevated significantly in the *FLG*-mutant group, and the TIDE score (Figure 2D, *p* = 0.023) was decreased compared with the non-mutant group. These data support that the patients with *FLG* mutation may benefit from targeted therapies and immunotherapy. In addition, combining tumor biological characteristics with somatic mutational signatures, the Sanger signatures decomposed 96 spectrums of mutational signatures into 30 different local areas (Alexandrov et al., 2013). A significant change occurred in signatures 1, 6, and 17 in the *FLG*-mutant group (Figures 2E,F). We further analyzed the effects of *FLG* gene mutations on immunologic characteristics of TCGA-STAD patients. The immune and stromal scores were used to quantify the immune and matrix components in STAD. The results showed that there was no significant difference in the immune (Figure 3A, *p* = 0.622) and stromal scores (Figure 3B, *p* = 0.504) among patients with *FLG* gene mutations, compared to patients without *FLG* gene mutations. Meanwhile, based on the TIMER database searches, we observed effects of somatic copy number alterations (SCNAs) on immune cell infiltration in *FLG* mutation tumor samples. The results suggested SCNAs, especially arm-level gain and high amplification, have a differential effect on tumor infiltrating immune cells including B cells, CD4^+^ T cells, CD8^+^ T cells, macrophages, neutrophils, and dendritic cells. Then, we further analyzed the correlation between *FLG* mutations and different biological pathways. The data demonstrated that changes in biological pathways were mainly enriched in cell cycle, DNA damage repair, DNA damage response, DNA damage replication, Fanconi anemia, homologous recombination, mismatch repair, and nucleotide excision repair (Figures 3D–K, *p* < 0.05).

### Drug sensitivity analysis

**FIGURE 4:**
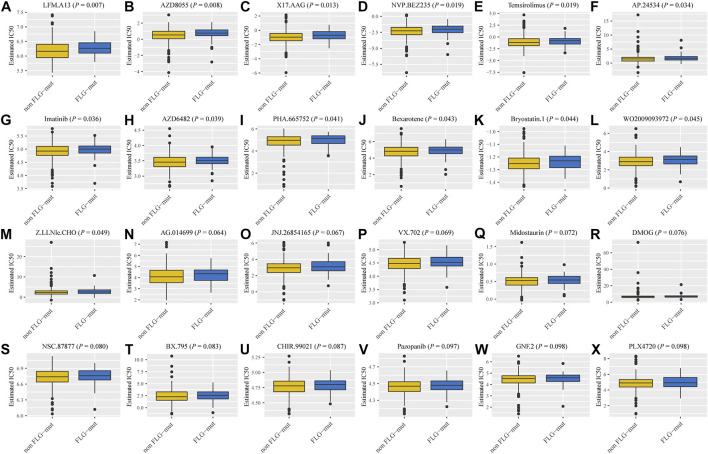
Sensitivity of *FLG* mutations on chemotherapeutic drugs and small-molecule anticancer drugs based on GDSC database analysis. **(A–X)** The IC50 values of 24 drugs between *FLG* and non-mutant patients.

To detect the drug sensitivity of *FLG* gene mutations in STAD patients, we utilized the Genomics of Drug Sensitivity in Cancer (GDSC) database, which contains drug sensitivity data for 138 chemotherapy drugs and small-molecule drugs. The results showed that the IC50 values of 24 chemotherapeutic drugs and small-molecule anticancer drugs were significantly different between *FLG* and non-mutant patients (*p* < 0.001, Figure 4), especially in LFM. A13, AZD8055, and X17. AAG.

### Construction and evaluation of the nomogram model

**FIGURE 5:**
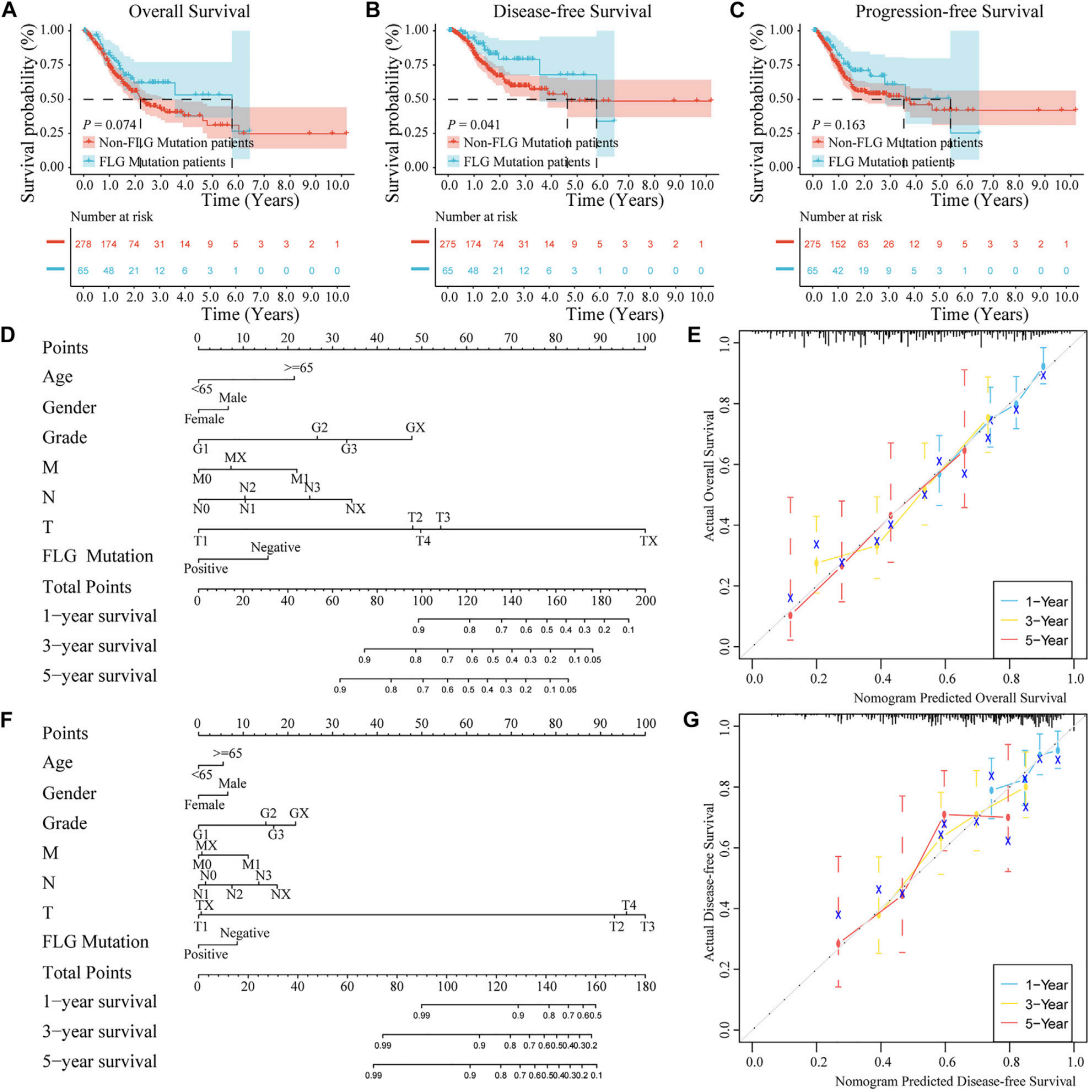
Effects of *FLG* gene mutation on clinicopathological features in the TCGA-STAD dataset. **(A–C)** In the TCGA-STAD dataset, the survival analysis showed that OS (log-rank *p* = 0.074) and DFS (log-rank *p* = 0.041) were better in patients with *FLG* gene mutations, but no significant impact on PFS (log-rank *p* = 0.163). **(D)** OS nomogram was constructed by the mutation of *FLG* gene combining with clinicopathological features. **(E)** Calibration curve of the *FLG* gene mutation nomogram. The abscissa axis is predicted survival, the ordinate axis is observed survival, every calculation is repeated 1,000 times. The calibration plots demonstrate good agreement between the predictions made by the nomogram and actual prognosis of patients for 1-,3-, and 5 years. **(F)** Nomogram constructed by the mutation of *FLG* gene combining with clinicopathological features. **(G)** Calibration curve of the nomogram of *FLG* gene mutation. The abscissa axis is predicted survival, the ordinate axis is observed survival, every calculation is repeated 1,000 times. The calibration plots demonstrate good agreement between the predictions made by the nomogram and actual prognosis of patients for 1-,3-, and 5 years.

To further explore the relationships between the *FLG* mutation status and clinical phenotype in bladder cancer, clinical correlation analysis of *FLG* gene mutations was conducted. In the TCGA-STAD dataset, the results of survival analysis showed that the *FLG* mutations suggest better overall survival (OS, log-rank *p* = 0.074, Figure 5A, Table 2) and disease-free survival (DFS, log-rank *p* = 0.041, Figure 5B, Table 3) in STAD patients, but have no significant effect in progression-free survival (PFS, log-rank *p* = 0.163; Figure 5C). To further explore the effects of mutations in *FLG* genes on TCGA-STAD patients, combined with the clinicopathological features of the patients, the univariate and multivariate Cox regression analyses showed that the mutation levels of the *FLG* gene are protective factors for STAD patients, but not independent protective factors, suggesting the potential diagnostic roles of *FLG* in STAD. Using clinicopathological parameters including age, gender, tumor stage, depth of invasion, lymph node metastasis, and distant metastasis, a nomogram to prognosticate OS and DFS was proposed and internally validated (Figures 5D,F). Discrimination of the nomogram was measured by calculating the c-index (concordance index), which indicated a high discrimination ability (OS: 0.675, 95% CI, 0.628–0.722; DFS: 0.663, 95% CI, 0.600–0.725). The calibration plot showed excellent concordance for the 1-, 3-, and 5-year predicted and actual OS and DFS probabilities (Figures 5E,G). Therefore, The *FLG* mutation can be used as an independent prognostic indicator in STAD. The prognostic nomogram based on *FLG* mutation may serve as a reliable model for predicting survival of patients.

**TABLE 2:** Univariate and multivariate analyses with the Cox proportional hazards regression model based on *FLG* gene mutation for predicting OS in TCGA database.

	Univariate cox analysis				Multivariate cox analysis			
	HR	HR.95L	HR.95H	*p*-value	HR	HR.95L	HR.95H	*p*-value
Age (≥65 vs. < 65)	1.55	1.09	2.20	0.013733	1.81	1.27	2.59	0.001054
Gender (male vs. female)	1.21	0.84	1.73	0.300951	1.25	0.87	1.79	0.229855
Grade (G3 & GX vs. G1 & G2)	1.45	1.02	2.05	0.03755	1.34	0.92	1.94	0.122587
T-stage (T3 & T4 & TX vs. T1&T2)	1.74	1.14	2.65	0.010536	1.53	0.99	2.37	0.054883
M-stage (M1 & MX vs. M0)	1.77	1.08	2.90	0.024188	1.86	1.13	3.07	0.014555
N-stage (N2 & N3 & NX vs. N0 & N1)	1.66	1.19	2.31	0.002856	1.47	1.04	2.08	0.029323
*FLG* mutation (MT vs. WT)	0.66	0.42	1.04	0.075563	0.70	0.44	1.13	0.144729

**TABLE 3:** Univariate and multivariate analyses with the Cox proportional hazards regression model based on *FLG* gene mutation for predicting DFS in TCGA database.

	Univariate cox analysis				Multivariate cox analysis			
	HR	HR.95L	HR.95H	*p*-value	HR	HR.95L	HR.95H	*p*-value
Age (≥65 vs. <65)	1.09	0.71	1.68	0.690387	1.31	0.84	2.03	0.231999
Gender (male vs. female)	1.53	0.94	2.48	0.088376	1.50	0.92	2.45	0.101933
Grade (G3 & GX vs. G1 & G2)	1.51	0.96	2.37	0.073327	1.23	0.77	1.99	0.387691
T-stage (T3 & T4 & TX vs. T1&T2)	2.12	1.19	3.77	0.010885	1.73	0.96	3.13	0.069976
M-stage (M1 & MX vs. M0)	1.67	0.86	3.23	0.12934	1.68	0.87	3.27	0.124968
N-stage (N2 & N3 & NX vs. N0 & N1)	2.09	1.35	3.22	0.000851	1.81	1.16	2.84	0.009531
FLG mutation (MT vs. WT)	0.52	0.28	0.98	0.044748	0.59	0.31	1.14	0.117505

### Differential expression analysis

**FIGURE 6:**
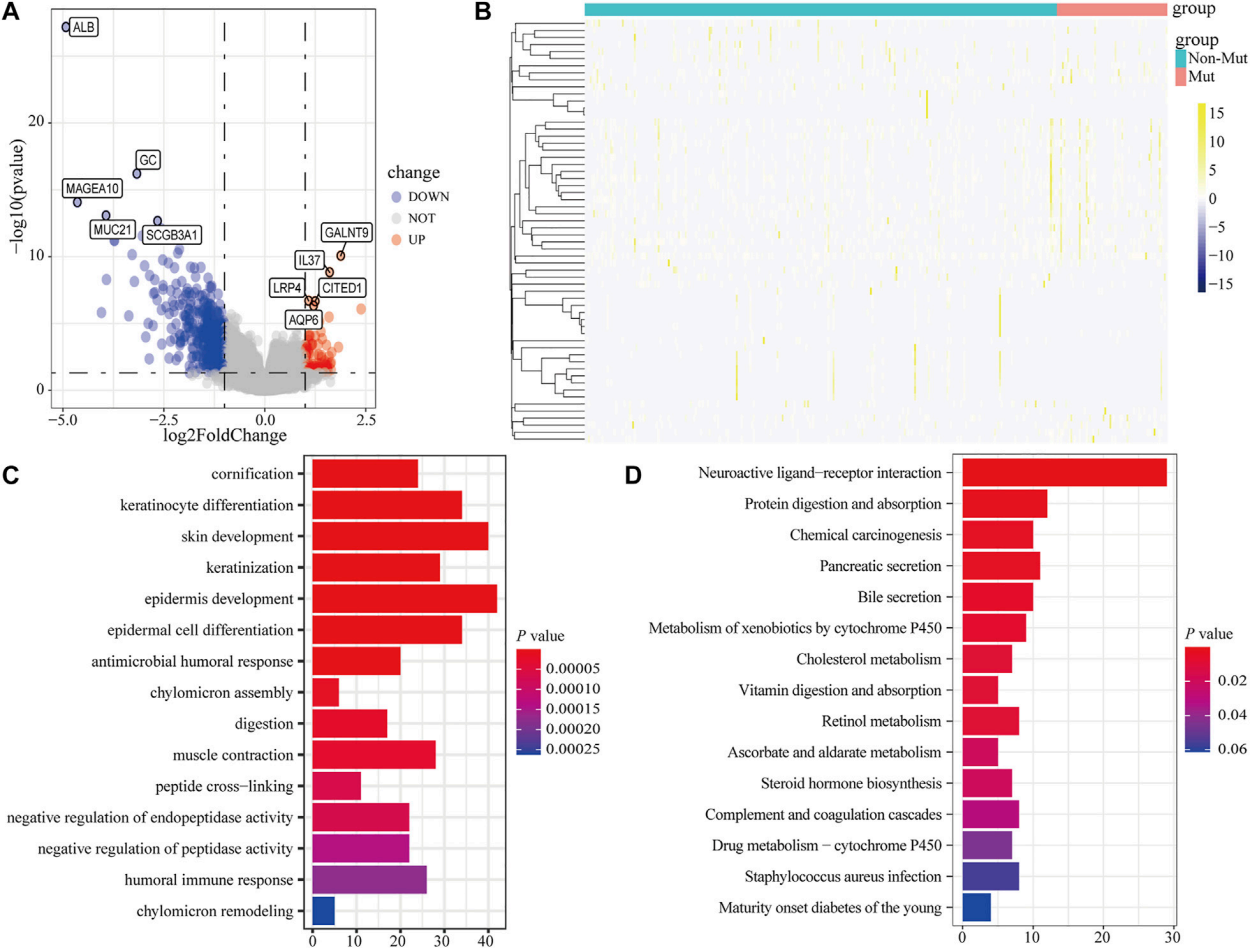
Functional enrichment analysis of the differentially expressed genes based on *FLG* gene mutation. **(A,B)** Volcano plot and heatmap show the expression of DEGs between *FLG*-mutant and non-mutant groups. **(C)** Based on CC, BP, and MF levels, GO analysis suggests that differentially expressed genes are closely related to cornification, keratinocyte differentiation, skin development, endoplasmic reticulum lumen, and cornified envelope biological processes. **(D)** KEGG analysis showed that these differentially expressed genes were participated in the neuroactive ligand–receptor interaction, protein digestion and absorption, chemical carcinogenesis, and pancreatic secretion and other biological related signaling pathways.

**FIGURE 7:**
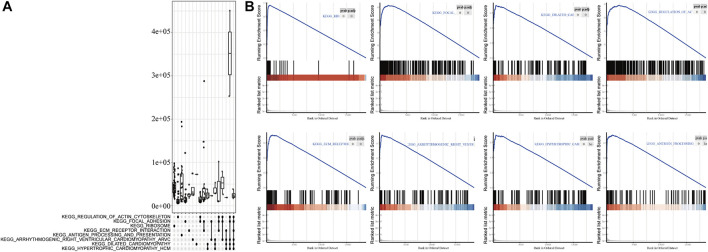
GSEA analysis based on differential expression of TCGA-STAD datasets. **(A)** Upper panel showed the GSEA analysis based on the differential expression of TCGA-STAD datasets. **(B)** Results showed tumor tissue with *FLG* mutation is closely related to ribosome, focal adhesion, dilated cardiomyopathy, regulation of action cytoskeleton, and ECM receptor interaction pathways.

To analyze the effect of *FLG* gene mutation on tumorigenesis in TCAG-STAD patients, the patients were divided into the *FLG*-mutant group and non-mutant group, and the differential gene expression analysis was further performed. After standardization and removal of batch effects in the microarray results, we found that 100 genes were significantly upregulated and 414 genes were significantly downregulated in the TCGA-STAD dataset (Figures 6A,B). To explore how *FLG* gene mutation may affect the gastric carcinogenesis, we conducted a functional enrichment analysis on the differentially expressed genes. GO analysis revealed a number of biological processes affected by *FLG* gene mutation, such as cornification, keratinocyte differentiation, skin development, endoplasmic reticulum lumen, and cornified envelope (Figure 5C, Table 4). The KEGG pathway analysis showed that the differentially expressed immune genes were related primarily to neuroactive ligand–receptor interaction, protein digestion and absorption, chemical carcinogenesis, and pancreatic secretion pathways (Figure 5D, Table 5). Next, we analyzed the functional enrichment pathways of the *FLG*-MT and *FLG*-WT groups in the TCGA-gastric cancer by GSEA (Table 6). The results showed that pathways enriched among the differentially expressed genes in *FLG*-mutant tissues, which include ribosomes, focal adhesion, dilated cardiomyopathy, regulation of actin cytoskeleton, and ECM receptor interaction, as is shown in Figure 7B.

**TABLE 4:** Gene Ontology analyses using differentially expressed genes (DEGs).

Ontology	ID	Description	Count	*p-* value	Gene
BP	GO:0070268	Cornification	24	1.96E-16	TGM1/KRT4/KLK5/KRT6C/KRT14/KRT13/SPINK5/KRT78/KRT5/KRT6A/KRT24/IVL/LCE3D/SPRR2F/SPRR3/KLK13/KRT6B/SPRR1A/SPRR2E/SPRR2B/SPRR2G/SPRR2D/KRT82/KRT38
BP	GO:0030216	Keratinocyte differentiation	34	1.05E-13	TGM1/KRT4/KLK5/KRT6C/KRT14/KRT13/SPINK5/KRT78/KRT5/KRT6A/S100A7/KRT24/SCEL/IVL/LCE3D/CERS3/SPRR2F/SPRR3/KLK13/KRT6B/SPRR1A/SERPINB13/SPRR2E/SPRR2B/LCE3E/LELP1/SPRR2G/KRTAP13-1/KRTAP2-3/KRTAP4-6/KRTAP11-1/SPRR2D/KRT82/KRT38
BP	GO:0043588	Skin development	40	1.17E-13	TGM1/KRT4/KLK5/KRT6C/KRT14/KRT13/LRP4/SPINK5/KRT78/DKK4/KRT5/COMP/KRT6A/S100A7/KRT24/SCEL/IVL/LCE3D/CERS3/SPRR2F/SPRR3/KLK13/DKK1/KRT6B/SPRR1A/SERPINB13/SPRR2E/SPRR2B/DACT2/FOXE1/LCE3E/LELP1/SPRR2G/KRTAP13-1/KRTAP2-3/KRTAP4-6/KRTAP11-1/SPRR2D/KRT82/KRT38
CC	GO:0005788	Endoplasmic reticulum lumen	30	7.19E-11	ALB/AFP/FGG/APOB/APOA1/VTN/GHRL/ITIH2/FGA/CHGB/CASQ2/BPIFB2/APOA4/SERPINA10/CES1/VGF/AHSG/COL2A1/CASQ1/PENK/COL26A1/F2/NOTUM/AMELX/COL9A3/APOA2/MTTP/PDIA2/GCG/SPP2
CC	GO:0001533	Cornified envelope	13	3.43E-09	TGM1/SCEL/IVL/LCE3D/SPRR2F/SPRR3/SPRR1A/SPRR2E/SPRR2B/LCE3E/LELP1/SPRR2G/SPRR2D
CC	GO:0062023	Collagen-containing extracellular matrix	31	1.23E-08	FGG/APOA1/VTN/ITIH2/ANXA8/ADIPOQ/FGA/APOC3/APOA4/CBLN1/NCAM1/AHSG/COL2A1/ORM1/SERPINA3/FGB/PTPRZ1/L1CAM/COMP/COL26A1/ORM2/F2/THBS4/S100A7/AMELX/COL9A3/PRTN3/MMP8/S100A8/PRSS1/SPP2
MF	GO:0048018	Receptor–ligand activity	41	2.88E-12	SCGB3A1/APOA1/GHRL/IL37/SCT/ANGPTL3/ADIPOQ/CHGB/FGF20/IGF2/SST/VIP/NTS/VGF/CARTPT/IL36A/DEFB4A/PENK/FGF19/F2/NTF4/THBS4/AMELX/PPBP/TTR/IFNW1/EPHA7/FGF3/INHA/EPGN/CSH2/NPPB/DKK1/GCG/LEFTY1/IFNL2/MLN/IFNL3/IL3/SLURP1/INSL5
MF	GO:0030546	Signaling receptor activator activity	41	4.01E-12	SCGB3A1/APOA1/GHRL/IL37/SCT/ANGPTL3/ADIPOQ/CHGB/FGF20/IGF2/SST/VIP/NTS/VGF/CARTPT/IL36A/DEFB4A/PENK/FGF19/F2/NTF4/THBS4/AMELX/PPBP/TTR/IFNW1/EPHA7/FGF3/INHA/EPGN/CSH2/NPPB/DKK1/GCG/LEFTY1/IFNL2/MLN/IFNL3/IL3/SLURP1/INSL5
MF	GO:0005179	Hormone activity	18	8.70E-10	GHRL/SCT/ADIPOQ/CHGB/IGF2/SST/VIP/NTS/VGF/CARTPT/PENK/TTR/INHA/CSH2/NPPB/GCG/MLN/INSL5

**TABLE 5:** Kyoto Encyclopedia of Genes and Genomes pathway enrichment analysis using differentially expressed genes.

ID	Description	Count	*p*-value	Gene
hsa04080	Neuroactive ligand–receptor interaction	29	1.92E-08	51738/6343/2862/6750/7432/4922/4886/155/5179/2147/6865/1136/2904/1443/5644/1129/886/2641/6863/2740/11255/2834/9248/4295/134864/3350/3361/57152/10022
hsa04974	Protein digestion and absorption	12	1.56E-05	1280/5222/4311/136227/1299/477/5644/10136/1358/643834/1357/643847
hsa05204	Chemical carcinogenesis	10	6.05E-05	1576/1543/1646/79799/127/54578/7367/6822/131/221357
hsa04972	Pancreatic secretion	11	7.31E-05	6343/1811/22802/3778/477/5644/886/10136/1358/9635/1357
hsa04976	Bile secretion	10	0.000121	6343/1576/79799/9971/477/570/54578/7367/6822/6555
hsa00980	Metabolism of xenobiotics by cytochrome P450	9	0.000198	1576/1543/79799/127/54578/7367/6822/131/221357
hsa04979	Cholesterol metabolism	7	0.000309	338/335/27329/345/337/336/8435
hsa04977	Vitamin digestion and absorption	5	0.000346	338/335/337/5948/9227
hsa00830	Retinol metabolism	8	0.000393	1576/1543/79799/127/9227/54578/7367/131

**TABLE 6:** Results of gene set enrichment analysis (GSEA).

Name	Size	Enrichment score	NES	*p-*value	Leading edge
KEGG_RIBOSOME	87	0.956547	1.656318	1.00E-10	Tags = 85%, list = 3%, signal = 83%
KEGG_FOCAL_ADHESION	199	0.830965	1.46027	2.21E-10	Tags = 39%, list = 8%, signal = 36%
KEGG_DILATED_CARDIOMYOPATHY	90	0.87904	1.521094	4.21E-07	Tags = 17%, list = 3%, signal = 16%
KEGG_REGULATION_OF_ACTIN_CYTOSKELETON	212	0.78167	1.373872	4.26E-07	Tags = 36%, list = 13%, signal = 32%
KEGG_ECM_RECEPTOR_INTERACTION	83	0.883822	1.530104	5.20E-07	Tags = 42%, list = 6%, signal = 40%
KEGG_ARRHYTHMOGENIC_RIGHT_VENTRICULAR_CARDIOMYOPATHY_ARVC	74	0.894349	1.542548	9.25E-07	Tags = 23%, list = 3%, signal = 22%
KEGG_HYPERTROPHIC_CARDIOMYOPATHY_HCM	83	0.874796	1.514478	2.17E-06	Tags = 17%, list = 3%, signal = 16%
KEGG_ANTIGEN_PROCESSING_AND_PRESENTATION	80	0.887199	1.532384	2.31E-06	Tags = 34%, list = 6%, signal = 32%

### Construction of PPI and ceRNA networks

**FIGURE 8:**
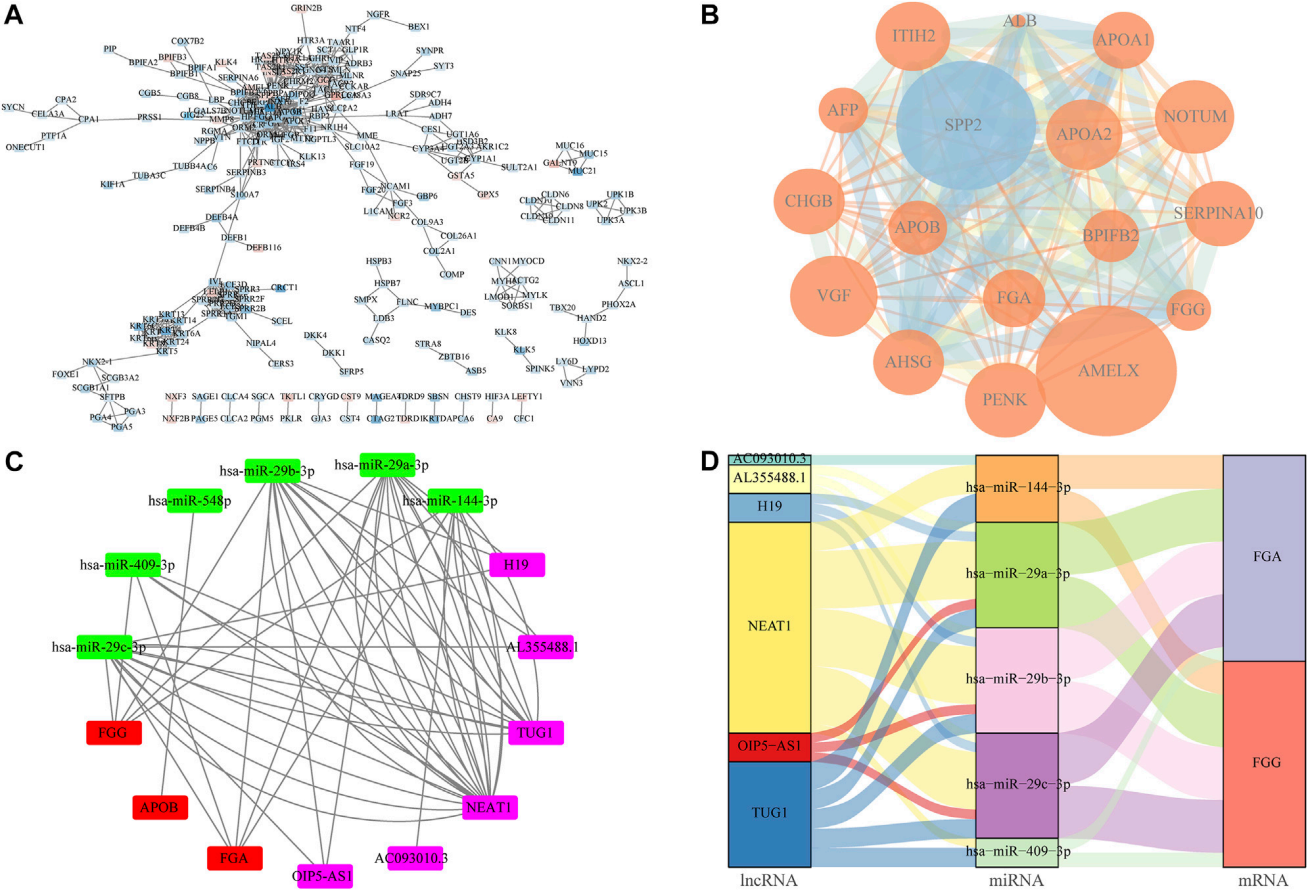
Construction of protein–protein interaction (PPI) and ceRNA network. **(A)** Layout in Figure for protein–protein interactions were created by Cytoscape software *via* the link provided by STRING. Values on the lines connecting the spheres indicate upregulated genes in red and downregulated genes in blue. The color shades have a positive relation with logFC. **(B)** MCODE algorithm is used to identify high density regions from the PPI network and the size of the circles is proportionate to the logFC, the color shades are proportional to *p*-value. **(C,D)** ceRNA interaction network was constructed by Cytoscpe and Sankey diagram based on the hub genes.

The PPI network of the DEGs was constructed using Cytoscape software based on the STRING database (Figure 8A). The upregulation of gene expression was indicated in red, whereas the downregulation of gene expression was indicated in blue. The MCODE plugins were used to choose the local high density as important nodes, and defined as hub genes (Figure 8B). The top six miRNAs with the highest number of target mRNAs are identified, hsa-miR-29c-3p, hsa-miR-409-3p, hsa-miR-548p, hsa-miR-29b-3p, hsa-miR-29a-3p, and hsa-miR-144-3p (Figure 8C). Also, by integrating the miRNA–mRNA and miRNA–lncRNA regulatory relationships, the ceRNA network of miRNA–mRNA–lncRNA interactions was constructed based on the miRTarBase. As shown in Figure 8D, the lncRNA–miRNA–mRNA network comprises six lncRNA, 5 miRNA, and 2 mRNA nodes.

### Discussion

Stomach adenocarcinoma (STAD) is among the most lethal human malignancies with both high mortality and high metastatic capacity (Smyth et al., 2020). Certain genes have been shown to play essential roles in STAD development (Wang et al., 2019). Despite the large number of studies carried out to date, our understanding of molecular mechanisms of STAD is still limited due to lack of stable and effective biomarkers. Therefore, there is an urgent need for more reliable molecular biomarkers, early diagnosis, and effective treatments. We collected the TCGA-gastric cancer containing clinical data and mutation data to explore the gene mutation and pathological mechanism of STAD using bioinformatic analysis, and we found that *FLG* gene mutations have an important impact on the clinical and biological characteristics of STAD. These mutations comprise missense and CNV changes, the majority being missense mutations. We further identified 100 upregulated DEGs and 414 downregulated DEGs in the *FLG* mutation group. We applied three pathway analyses, GO, KEGG, and GSEA, to analyze the biological functions of these DEGs. Go analysis revealed that changes in modules were mostly enriched in biological processes such as keratinization, keratinocyte differentiation, and skin development. KEGG pathway enrichment analysis reveals that DEGs were mainly involved in neuroactive ligand–receptor interaction, protein digestion and absorption, and chemical carcinogenesis pathways. GSEA also revealed biologically relevant pathways associated with ribosome, focal adhesion, dilated cardiomyopathy, regulation of actin cytoskeleton, and ECM receptor interaction. Then through PPI network construction, key hub genes and the most significant module were selected. In addition, we found that mutations in the *FLG* gene were associated with immune cell infiltration, and genetic mutation of *FLG* in STAD causes increased sensitivity to anticancer agents. We examined the relationship between *FLG* mutations and prognosis. *FLG* mutations were significantly associated with better disease-free and overall survival, and appeared to be an independent prognostic factor. Based on these findings, we proposed that the *FLG* gene could be regarded as a potential biomarker to further explore the molecular mechanism and the prognostic effects of STAD.

Gastric cancer development involves multiple gene alterations. In this study, we first applied computational algorithms to detect driver genes using somatic mutations of STAD tissues and classified the data into different categories. *TTN*, *TP53*, *MUC16*, *ARIDIA*, *LRPIB*, *SYNE1*, *FLG*, *CSMD3*, *FAT4*, and *PCLO* were the 10 most frequently mutated genes, which is partially in concordance with previously published studies (Wang et al., 2020). For example, *TP53* mutations were more common in gastrointestinal adenocarcinomas with intact DNA mismatch repair protein expression (Krishnamurthy et al., 2022). *MUC16* mutations were found to be potentially associated with GC prognosis, some mutation statuses of *MUC16* and *TTN* were identified with high potential in predicting TMB (Yang et al., 2020). For another example, the somatic mutation rate of the *ARIDIA* gene varies significantly between GC patients of Asian and Caucasian descent (20.7% vs. 32.1%, *p* = 0.01), which might have important implications for precise therapeutics in GC patients (Jia et al., 2017). There are also genes, such as *SYNE1*, the high level of *SYNE1* promoter methylation was associated with poorer chemotherapy efficacy in advanced gastric cancer patients (Qu et al., 2021). Also, *FAT4*, a tumor suppressor gene exerts an important role in cell adhesion. Reduced expression of *FAT4* and increased methylation of its promoter may accelerate the progression of benign tumors to malignant GC (Pilehchian et al., 2017). However, at present, there are still limited studies on the effects of *FLG* mutations on STAD. We, therefore, chose the *FLG* gene for subsequent studies.

The protein encoded by the *FLG* gene is an intermediate filament-associated protein that aggregates keratin intermediate filaments in mammalian epidermis. It is initially developed as profilaggrin, which is localized in keratohyalin granules, and is subsequently proteolytically processed into individual functional filaggrin molecules. In humans, the *FLG* gene is located within the epidermal differentiation complex (EDC) on chromosome 1q21, spans∼25 kb of DNA and comprises three exons and two introns. Exon 1 is non-coding and protein translation start site begins within exon 2. The majority of the profilaggrin protein is encoded by the exon 3 (Sandilands et al., 2009). The *FLG* gene is composed of tandem repeats with CNVs consisting of 10, 11, or 12 copies of the sequence encoding filaggrin monomers. Tandem repeats are usually present in coding and regulatory regions of the human genomes, so it has a greater chance of mutation and are associated with many genetic diseases (Kim et al., 2020). The loss-of-function mutations in *FLG* are common, and approximately 2–10% of Europeans carried at least one *FLG* null mutation. The *FLG* loss-of-function mutation is associated with ichthyosis vulgaris, atopic dermatitis, inflammatory dermatosis, and inflammation dysregulated diseases such as asthma and allergy (Brown and McLean, 2012; Zhu et al., 2018). One previous study has explored two single-nucleotide polymorphism (SNP) loci of the *FLG* gene, rs3126085 and K4671X, which were associated with EBV-associated gastric carcinoma for (EBVaGC). The genotype AA of rs3126085 (c.3321delA), as a most popular *FLG* mutation in Chinese Han people, was considered as a hazardous sign for EBV-associated gastric carcinoma (Yang et al., 2017). Another study detected the *FLG* rs2065955 genotype and allele distribution in 64 EBV-associated gastric carcinoma samples, and found genotype CC may contribute more to the risk of developing EBVaGC (Kuang et al., 2016). Nevertheless, no significant difference in *FLG* expression was detected by immunohistochemical analysis in those studies. It would be speculative to guess that a single-nucleotide variation in intron may have little impact on the gene expression, or the intragenic CNV may also complicate the process of cancer pathogenesis. Interpretation of the results of prior studies may have been hampered by limited sample sizes and heterogeneous study populations. So, it is necessary to integrate the complexity of cancer genome data and multiplex optimization in a single method as bioinformatics to facilitate data integration and processing.

We found missense mutation is the predominant form of *FLG* genetic variation through the analysis of the amino acid changes in the *FLG* mutation group. Next, we demonstrated the existence of CNV alternations in the *FLG* mutation group, which would actually alter the *FLG* gene structure and function of tumor cells, so further analysis of these CNV regions should be treated as priority in the future. We finally identified 100 upregulated DEGs and 414 downregulated DEGs. The heatmap of all DEGs showed obvious difference between the *FLG*-mutant and non-mutant groups. To better understand the interactions among DEGs, we further performed functional enrichment analysis. The GO term enrichment analysis shows that DEGs were mainly involved in biological processes such as cornification, keratinocyte differentiation, skin development, endoplasmic reticulum lumen, and cornified envelope. As in previous studies, the role of filaggrin in skin physiology and disease is well-established; we thus surmise that *FLG* gene mutation may also have a role in epidermal differentiation, morphogenesis, and homeostasis of gastric cancer cells. These signatures may help to untangle remaining questions about the biological processes in STAD progression.

TMB and MSI can serve as the predicting factors for selecting patients that likely to benefit from immune checkpoint inhibition therapy. Notably, the mutant *FLG* expression was correlated with high TMB and MSI values. In addition, the patients in the *FLG*-MT group had a negative TIDE score, indicating lack of tumor immune evasion phenotypes. Although we did not find the differences in the immune score and stromal score in the *FLG* mutation group, but at different mutation levels, we observed significant changes in the immune cell infiltration. Filaggrin is a key protein involved in many inflammation dysregulated diseases, and lack of filaggrin protein can induce inflammation and T-cell infiltration (Brown and McLean, 2012). Our analysis also found that cell cycle and DNA repair–related pathways showed significant enrichment in the *FLG* mutation group. We have validated the performance of *FLG* mutation using the GDSC heterogeneous dataset containing molecular descriptors of drugs with transcriptomic expressions of STAD cell lines. We screened 138 drugs and compared the response to common anticancer drugs between the *FLG* mutation and non-mutation groups and identified significantly different responses to three of these drugs. The application of the Bruton’s tyrosine kinase (BTK) inhibitor LFM-A13 in solid cancer has been discovered recently. It promotes apoptosis, has an antiproliferative effect, and increases the sensitivity of cancer cells to chemotherapy drugs (Uckun et al., 2011). AZD8055 is a small-molecule inhibitor of mammalian target of rapamycin (mTOR) kinase activity; mTOR plays an important, albeit complex, role in tissue homeostasis and tumorigenesis (Marshall et al., 2011). The positive expression of p-mTOR was more frequent in advanced gastric cancers (Murayama et al., 2009).

KEGG pathway enrichment analysis reveals that DEGs were mainly involved in several pathways such as neuroactive ligand–receptor interaction, protein digestion and absorption, and chemical carcinogenesis. The neuroactive ligand–receptor interaction pathway was suggested to play a key role in the effect of DNA methylation on GC prognosis (Dai et al., 2021). The protein digestion and absorption pathway was found to be significantly enriched in GC tissues (Liu et al., 2020). In particular, the loss of *FLG* in neck squamous cell carcinoma tissue was found to result in a dramatic resistance to targeted therapies (Bai et al., 2021). GSEA analysis further revealed significant enrichment of ribosome, focal adhesion, dilated cardiomyopathy, regulation of actin cytoskeleton, and ECM receptor interaction in mutant *FLG* tissues. Actin cytoskeletal remodeling was proven to affect epithelial–mesenchymal transition in gastric cancer cells (Yang et al., 2017). Subsequently, in order to find the co-existence pattern among all the DEGs, we constructed the PPI network. From the network, several hub genes with high degrees were found. For example, the G-protein–coupled receptor *GPRC6A* is located in multiple tissues, including gastrointestinal epithelia; studies have shown that *GPRC6A* is involved in regulating glucose and fat metabolism in certain cancers such as prostate cancer progression (Pi et al., 2021). *GALNT9* (an initiator of O-glycosylation) is a member of a sub family that differs significantly in the sequence from other GALNAC-T members, and has been shown to be dysregulated in cancer by promoter methylation (Pangeni et al., 2015).

This was the first time we evaluated the relationship between *FLG*, clinicopathological features, and prognosis in STAD. We explored the prognostic value of *FLG* mutation in the TCGA-STAD database. Compared to the non-mutant group, the results showed that patients with mutant *FLG* had better overall survival and disease-free survival, but had no effect on progression-free survival. Combining *FLG*-mutant tumor types with clinicopathological features showed that *FLG* mutation was a protective factor but not an independent protective factor. Our current nomogram also showed that the *FLG* mutation had a significant influence on 1-, 3-, and 5-year prognosis, future studies are needed to externally validate the proposed nomograms to establish their value in predicting the long-term prognosis of STAD patients.

A few limitations of the current study are worth mentioning. Although microarray-based bioinformatic analysis is a powerful analytic tool for a deeper understanding of molecular mechanisms and for identifying potential biomarkers of STAD, further efforts are needed. First, though the *FLG* gene has been demonstrated to have a high diagnostic and prognostic value in STAD patients, current understanding of detailed mechanisms is limited. For deeper understanding of mechanisms underlying the *FLG* gene functions, the mRNA expression levels of the *FLG* gene need to be performed by RT-PCR, and the protein expression levels of the *FLG* gene need to be performed by Western blot and the immunochemistry method in separate cohorts. Moreover, a more detailed examination by utilizing a combination of *in vitro* and *in vivo* techniques may further elucidate the diagnostic and therapeutic effects of the *FLG* gene in STAD patients. Second, as a retrospective study, potential selection bias and recall bias were inevitable, and more studies in settings with better statistics are necessary. Third, due to the incomplete clinical information on STAD from the TCGA database and the limited sample size, we need more long-term follow-up data and the clinical benefit of early detection for further validation and study.

## Conclusion

Our study first comprehensively demonstrated the expression and function, and prognostic value of the *FLG* gene in STAD. We found that the *FLG*-MT group showed a higher mutation load and immunogenicity in STAD patients. We further identified DEGs between the *FLG*-WT and *FLG*-MT groups and performed GO analysis, pathway enrichment analysis, PPI network construction, and prognostic analysis to understand its role in STAD at the molecular level. Further analyses showed that the DFS and OS were significantly different by mutation status and *FLG* gene mutation might be a protective factor. We also demonstrated that *FLG* mutation patients showed comparably high mutation counts than *FLG* intact patients in DNA damage repairment-related pathways. Then we found that *FLG* mutations resulted in increased gastric cancer sensitivity to 24 chemotherapeutic drugs and small-molecule anticancer drugs, especially in LFM. A13, AZD8055, and X17. AAG. This study not only suggests the potential value of the *FLG* gene as a novel biomarker, but also offers new diagnostic and/or therapeutic avenues for STAD.

## Data Availability

The datasets presented in this study can be found in online repositories. The names of the repository/repositories and accession number(s) can be found in the article/Supplementary Material.
